# Selective cytotoxicity of solamargine via oxidative stress and caspase-independent mechanisms in human glioblastoma cells

**DOI:** 10.1007/s10637-026-01599-y

**Published:** 2026-02-05

**Authors:** Arthur Barcelos Ribeiro, Marcela de Melo Junqueira, Ricardo Andrade Furtado, Jairo Kenupp Bastos, Denise Crispim Tavares

**Affiliations:** 1 University of Franca, Avenida Dr. Armando Salles Oliveira, 201, 14404-600 Franca, São Paulo Brazil; 2 School of Pharmaceutical Sciences of Ribeirão Preto, University of São Paulo, Av. do Café s/n, 14040-903 Ribeirão Preto, São Paulo Brazil

**Keywords:** Glioma, Natural product, Glycoalkaloid, Temozolomide, Hypoxia, DNA damage

## Abstract

IDH-wild-type glioblastoma (IDH-wildtype GB) is an aggressive and genetically heterogeneous tumor characterized by intrinsic resistance to radiotherapy and chemotherapy, while surgical resection remains inherently limited by its diffuse infiltrative growth, leading to poor clinical outcomes. Natural products such as solamargine (SM), a steroidal glycoalkaloid with cytotoxic and antitumor properties, have emerged as potential adjuvant strategies. Here, we investigated the effects of SM on proliferation, clonogenic survival, morphology and migration of IDH-wildtype GB cell lines (U-87MG, U-251MG and T98-G) and non-tumoral astrocytes under normoxic and hypoxic conditions, as well as its interaction with temozolomide (TMZ). Under normoxia, SM reduced cell viability in a dose- and time-dependent manner, with IC₅₀ values between 5.04 and 9.53 μM and showed enhanced cytotoxicity under hypoxia. TMZ alone displayed modest activity, and its combination with SM produced predominantly antagonistic effects. Clonogenic assays confirmed the antiproliferative potential of SM, with significant inhibition of colony formation at 2.5 μM. SM induced marked morphological alterations but did not significantly impair migration in wound-healing assays. In U-87MG cells, SM triggered G₂/M cell-cycle arrest, increased intracellular reactive oxygen species generation, and elevated γH2AX protein expression, indicating oxidative stress-associated DNA damage. However, cleaved caspase-3 and p53 were not detected, suggesting a predominantly non-apoptotic mode of cell death. Together, these findings support SM as a promising candidate for IDH-wildtype GB therapy and underscore the need for further studies to clarify its mechanisms of action and optimize its therapeutic use.

## Introduction

IDH-wild-type glioblastoma (IDH-wildtype GB) is the most common and lethal primary malignant tumor of the adult central nervous system, classified as WHO grade 4 based on histological features and molecular criteria such as EGFR amplification, combined chromosome 7 gain/10 loss (+ 7/− 10), or TERT promoter mutation [1]. Maximal safe resection followed by radiotherapy with concomitant and adjuvant temozolomide (TMZ) remains the standard of care, but median overall survival is 15 months and only 5.8–10% of patients survive beyond five years [2]. This poor prognosis reflects intrinsic refractoriness driven by genetic heterogeneity, cellular plasticity, a hostile tumor microenvironment, limited drug penetration across the blood–brain barrier, and highly infiltrative growth [3].

Among these features, tumor hypoxia is a central driver of resistance: low oxygen levels in poorly perfused, perinecrotic areas stabilize HIF-1α and activate pro-angiogenic and metabolic programs (e.g., VEGF, CAIX, GLUT1), promoting invasion, immune suppression and reduced responses to radiotherapy and TMZ [4]. In parallel, overexpression of O^6^-methylguanine–DNA methyltransferase (MGMT), which removes TMZ-induced O^6^-methylguanine adducts, further compromises TMZ efficacy [2].

**Fig. 1:**
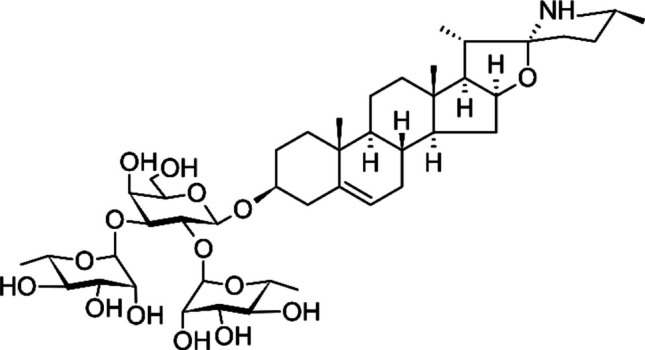
Chemical structure of solamargine

Natural products are a major source of anticancer drugs, accounting for 64.9% of newly developed antineoplastic agents [5]. In IDH-wildtype GB, several natural compounds exhibit antiproliferative and chemosensitizing effects by disrupting proliferative and metabolic signaling, enhancing apoptosis, and modulating epigenetic and DNA-repair mechanisms [6]. Within this context, solamargine (SM, Fig. 1), a steroidal glycoalkaloid from Solanum species, has emerged as a promising candidate: it shows broad cytotoxic and antiproliferative activity across tumor models and modulates multiple survival and stress-response pathways [7–9].

Despite this growing evidence, data on SM in IDH-wildtype GB, particularly in combination with TMZ, remain scarce. Considering that TMZ resistance is a major clinical barrier, we evaluated SM both as a direct antitumor agent and as a potential chemosensitizer capable of enhancing TMZ-induced cytotoxicity, suppressing proliferation, and modulating key resistance pathways. Accordingly, we investigated the effects of SM, alone and combined with TMZ, on IDH-wildtype GB cells in 2D culture, including under hypoxic conditions, assessing key cellular responses in vitro.

## Material and methods

### Chemical agents evaluated

Solamargine (SM; CAS 20311–51-7) was isolated and characterized as described by Munari [10]. For each experiment, SM was freshly weighed, dissolved in sterile deionized water, and diluted in complete medium to 2.5–20 µM. Temozolomide (TMZ; CAS 85622–93-1; Sigma-Aldrich) was prepared as a 100 mM stock in anhydrous Dimethyl sulfoxide (DMSO), aliquoted and stored at − 20 °C, protected from light. Immediately before use, aliquots were diluted in complete medium to 62.5–1500 µM; final DMSO did not exceed 1% (v/v) and was kept constant across conditions. Only clear, precipitate-free solutions were used and freeze–thaw cycles were avoided.

### Cell lines and culture conditions

Human IDH-wildtype GB cell lines U-87MG, U-251MG, and T-98G, and non-tumoral human astrocytes (NHA) were cultured in Dulbecco’s modified Eagle medium (DMEM; Sigma-Aldrich) supplemented with 10% (v/v) fetal bovine serum (Cultilab, Campinas, SP, Brazil), 1.2 g/L sodium bicarbonate (Sigma-Aldrich), 2.38 g/L HEPES (Sigma-Aldrich), 0.06 g/L penicillin (Sigma-Aldrich), and 0.10 g/L streptomycin (Sigma-Aldrich). Cells were maintained in T25 flasks (25 cm^2^ growth area) at 36.5 °C in a humidified atmosphere of 5% CO₂.

### Cell metabolic viability assay

The cytotoxicity of SM and/or TMZ under normoxia and hypoxia was assessed by the XTT assay (Roche), following the manufacturer’s instructions. NHA, U-87MG, U-251MG and T-98G were seeded in 96-well plates at 1 × 10^4^, 5 × 10^3^, 2.5 × 10^3^ and 1 × 10^3^ cells/well for 24, 48, 72 and 96 h assays, respectively. Plates included negative (untreated), vehicle (1% DMSO) and positive (25% DMSO) controls.

Under normoxia (36.5 °C, 5% CO₂), after 24 h attachment, cells were exposed to SM (2.5–20 µM) or TMZ (62.5–1500 µM) for 24–72 h. For combination experiments (total 96 h), cells were pretreated with SM (7.5–12.5 µM) for 24 h and then incubated with TMZ (750–1500 µM) for 72 h. At the end of treatment, wells were rinsed with PBS, XTT was added (0.3 mg/mL), and plates were incubated for 17 h at 36.5 °C.

For hypoxic assays (36.5 °C, 5% O₂, 5% CO₂, 90% N₂), IDH-wildtype GB cells were pre-adapted to hypoxic incubation for 24 h, then exposed to SM (2.5–20 µM) for an additional 24 h, followed by XTT incubation (0.3 mg/mL) for 17 h, resulting in a total of 65 h under hypoxic conditions. Throughout the experiment, cells were maintained in hypoxia, with only brief and unavoidable exposures to ambient conditions during plate handling (medium exchange, drug addition, and XTT application). After each manipulation, plates were promptly returned to the hypoxic incubator and sealed with gas-permeable membranes to maintain hypoxic conditions.

Absorbance was read at 450 nm with 620 nm reference. IC₅₀ values were calculated in GraphPad Prism; the selectivity index (SI) was defined as IC₅₀(NHA)/IC₅₀(IDH-wildtype GB). SM–TMZ interactions were analyzed by isobolography and the combination index (CI) in CompuSyn, with CI < 1, = 1, and > 1 indicating synergism, additivity, and antagonism, respectively [11].

### Clonogenic efficiency assay

The antiproliferative effect of SM was evaluated by clonogenic assay according to Franken [12]. NHA, U-87MG, U-251MG and T-98G were seeded in 6-well plates (3 × 10^2^ cells/well), allowed to adhere for 2 h (36.5 °C, 5% CO₂), and then exposed to SM (2.5–10 µM) for 24 h; negative and positive control groups (Methyl methanesulfonate, MMS, 400 µM) were included. After treatment, medium was replaced, and colonies were allowed to grow for at least six cell division cycles. Colonies were then fixed (methanol/acetic acid, 1:1), stained with Giemsa (1:20) and counted to calculate the surviving fraction as described by Franken [12].

### Cell migration assay

Cell migration was assessed by wound-healing assay [13]. U-87MG, U-251MG and T-98G were seeded in 6-well plates (5 × 10^5^ cells/well) and grown to confluence. A linear scratch was made with a 200 µL tip, and wells were washed with PBS. Cells were then incubated in serum-free medium with SM at 1, 2.5 and 3 µM (U-87MG, U-251MG) or 1, 2.5 and 5 µM (T-98G); a scratch-only control was included. Images were acquired at baseline and after 24 h (U-87MG, U-251MG) or 72 h (T-98G) using an inverted microscope with a digital camera (100 ×). Scratch closure was quantified in ImageJ and expressed as % closure [14].

### Cellular morphological assay by phase contrast microscopy

For morphological assessment, U-87MG, U-251MG and T-98G were seeded in 6-well plates (2 × 10^5^ cells/well), allowed to adhere for 24 h, and treated with SM (5–20 µM) or MMS (400 µM, positive control). Phase-contrast images (200 ×) were acquired at 5 min, 1 h and 24 h. Cell shape, cytoplasmic extensions, adhesion, density and proportion of detached cells were evaluated in a time- and concentration-dependent manner to monitor SM-induced cytotoxic changes.

### Evaluation of SM’s mechanism of action in U-87MG cells

Mechanistic studies focused on U-87MG, selected based on pronounced cytotoxic and antiproliferative responses. Exposure regimens were defined from prior assays (XTT IC₅₀ = 7.68 µM at 24 h; marked morphological changes at 20 µM for 1 h). Cells were seeded in 6-well plates (3 × 10^5^ cells/well), allowed to adhere for 24 h, and treated with SM at 20 µM for 1 h or 7.68 µM for 24 h. Untreated and appropriate positive controls were included in each assay.

#### Cell death assay

Cell death was quantified on a Muse® Cell Analyzer using the Annexin V & Dead Cell Kit, following the manufacturer’s instructions. After treatment, cells were stained with Annexin V-PE and 7-AAD for 20 min in the dark, and 10,000 events/sample were acquired. The percentages of viable (Annexin V⁻/7-AAD⁻), early apoptotic (Annexin V⁺/7-AAD⁻), late apoptotic/necrotic (Annexin V⁺/7-AAD⁺) and dead cells were determined.

#### Cellular morphological assay by fluorescence microscopy

Acridine orange/ethidium bromide (AO/EB) staining was performed as described by Kasibhatla [15]. After treatment, cells were incubated with AO/EB (100 µg/mL each) for 2 min in the dark, rinsed with PBS and immediately examined by fluorescence microscopy. Nuclear morphology, chromatin condensation/fragmentation and membrane integrity were qualitatively evaluated.

#### Cell cycle assay

Cell-cycle distribution was analyzed on the Muse® Cell Analyzer using the Muse® Cell Cycle Kit. After treatment, cells were fixed in cold 70% ethanol for 3 h at − 20 °C, stained with propidium iodide for 20 min in the dark, and at least 10,000 events/sample were acquired. The proportions of cells in G0/G1, S and G2/M phases were quantified from DNA-content histograms.

#### Oxidative stress assay

Intracellular reactive oxygen species (ROS) were measured using the Muse® Oxidative Stress Kit. After 24 h attachment, cells were treated with SM (7.68 or 20 µM) for 1 or 24 h. Untreated cells and H₂O₂ (400 µM) served as negative and positive controls. A minimum of 10,000 events/sample were acquired, and ROS-negative and ROS-positive populations were determined using kit-defined gates.

#### Protein expression analysis

To investigate SM-induced molecular changes, western blotting was performed in U-87MG cells. Cells were seeded in 75 cm^2^ flasks (1 × 10^6^ cells/flask), incubated for 24 h, and treated with 7.68 µM SM for 24 h; untreated and MMS-treated (1000 µM) cells were controls. Protein extraction, quantification, SDS–PAGE and transfer were performed as described by Ribeiro [16]. Equal protein amounts (60 µg/lane) were resolved, transferred to PVDF membranes, blocked and probed with antibodies against p53, phospho-H2A.X (Ser139) and cleaved caspase-3; β-actin was used as loading control. HRP-conjugated secondary antibodies were applied, and bands were detected within the linear range and quantified by densitometry in ImageJ. Target/β-actin ratios were normalized to untreated controls.

### Statistical analysis

Data were analyzed by ANOVA according to the experimental design. Cell death, cell cycle, oxidative stress, clonogenic and western blot data were evaluated by Two-Way ANOVA with Bonferroni’s or Tukey’s multiple comparisons test, as appropriate. Cell metabolic viability and migration assays were analyzed by One-Way ANOVA followed by Tukey’s test. Results are expressed as mean ± standard deviation, with α = 0.05. Analyses were performed using GraphPad Prism 6.0 (GraphPad Software, La Jolla, CA, USA).

## Results

### Differential cytotoxic profiles of SM and TMZ, alone and in combination, under normoxic and hypoxic conditions

SM reduced the viability of U-87MG, U-251MG and T98-G in a time-dependent manner under normoxia, with IC₅₀ values of 5.04–9.53 µM, always significantly lower than in NHA, indicating selective cytotoxicity (SI = 1.4–2.0, maximal in U-87MG: 2.0 at 24–48 h; 1.9 at 72 h). Under hypoxia, 24-h IC₅₀ values were 6.88 µM (U-87MG), 7.39 µM (U-251MG) and 6.75 µM (T98-G), all lower than the 24-h normoxic values, suggesting greater potency at low O₂. TMZ practically did not reduce the viability of tumor cell lines at 24–48 h (viability ≥ 95% up to 1500 µM, with no measurable IC₅₀). At 72 h, there was modest cytotoxicity in U-87MG (IC₅₀ = 1,069.28 µM) and U-251MG (IC₅₀ = 1,345.52 µM), whereas T98-G did not reach 50% inhibition (85.4 ± 1.4% viability at 1500 µM). NHA were more sensitive to TMZ, with 77.70 ± 3.87% viability at 1500 µM/24 h and IC₅₀ values of 1,274.96 µM (48 h) and 680.75 µM (72 h) (Table 1)*.*

**Table 1:** IC₅₀ values and SI for SM under normoxic and hypoxic conditions, and IC₅₀ values for TMZ in U-87MG, U-251MG and T98-G glioblastoma cell lines after 24, 48 and 72 h of treatment

Group	Cell line				IC_50_ (µM)			
		24 h			48 h		72 h	
		Normoxia	SI	Hypoxia	Normoxia	SI	Normoxia	SI
SM	NHA	15.36 ± 0.09	-	-	13.51 ± 0.23^b^	-	9.76 ± 0.08^bc^	-
	U-87MG	7.68 ± 0.15^ad^	2.0	6.88 ± 0.06^b^	6.55 ± 0.45^abe^	2.0	5.04 ± 0.45^abce^	1.9
	U-251MG	8.33 ± 0.08^a^	1.8	7.39 ± 0.09^b^	7.28 ± 0.67^ab^	1.8	5.52 ± 0.69^abc^	1.7
	T98-G	9.53 ± 0.17^a^	1.6	6.75 ± 0.26^b^	8.41 ± 0.88^ab^	1.6	6.95 ± 0.30^abc^	1.4
TMZ	NHA	> 1500	-	-	1274.96 ± 12.89^b^	-	680.75 ± 7.98^bc^	-
	U-87MG	> 1500	-	-	> 1500	-	1069.28 ± 6.07^bc^	-
	U-251MG	> 1500	-	-	> 1500	-	1345.52 ± 9.07^bc^	-
	T98-G	> 1500	-	-	> 1500	-	> 1500	-

*SM* – Solamargine; *TMZ* – Temozolomide; *NHA* – Normal human astrocyte; *U-87MG* – Human glioblastoma; *U-251MG* – Human glioblastoma; *T98-G*—Human glioblastoma. Values are mean ± standard deviation (*n* = 3)

^a^Significantly different from the non-tumoral cell line (*p* < 0.05)

^b^Significantly different from the 24-h treatment in normoxia condition (*p* < 0.05)

^c^Significantly different from the 48-h treatment in normoxia condition (*p* < 0.05)

^d^Significantly different from U-251MG and T98-G cell lines (*p* < 0.05)

^e^Significantly different from T98-G cell line (*p* < 0.05)

In SM + TMZ combinations, combination index (CI) values ranged from 1.1 to 1.9, indicating predominantly antagonistic interactions, which became more pronounced at higher concentrations. The strongest antagonism (CI = 1.9) occurred with 12.5 µM SM + 1500 µM TMZ in U-87MG and U-251MG. In these cell lines, the combinations did not outperform SM alone, although they were superior to TMZ alone, showing that the effect was predominantly driven by SM. T98-G showed a similar pattern. Thus, within the tested ranges, SM + TMZ offered no advantage over SM monotherapy (Table 2)*.*

**Table 2:** CI obtained in U-87MG, U-251MG and T98-G cell lines after 24 h of treatment with SM followed by 72 h of treatment with TMZ

SM	TMZ	Combination indexes		
(µM)	(µM)	U-87MG	U-251MG	T98-G
**7.5**	750	1.4	1.3	1.1
**10**		1.3	1.3	1.1
**12.5**		1.5	1.5	1.3
**7.5**	1000	1.6	1.7	1.8
**10**		1.4	1.5	1.4
**12.5**		1.7	1.6	1.4
**7.5**	1250	1.5	1.5	1.5
**10**		1.6	1.6	1.3
**12.5**		1.8	1.8	1.5
**7.5**	1500	1.6	1.5	1.4
**10**		1.7	1.7	1.4
**12.5**		1.9	1.9	1.6

Interpretation of Combination Index (CI): CI < 1 (synergistic), CI = 1 (additive), CI > 1 (antagonistic); *SM* – Solamargine; *TMZ* – Temozolomide; *NHA* – Normal human astrocyte; *U-87MG* – Human glioblastoma; *U-251MG* – Human glioblastoma; *T98-G*—Human glioblastoma, (*n* = 3)

### Effects of SM on clonogenic growth, migration, and cellular morphology

**Fig. 2:**
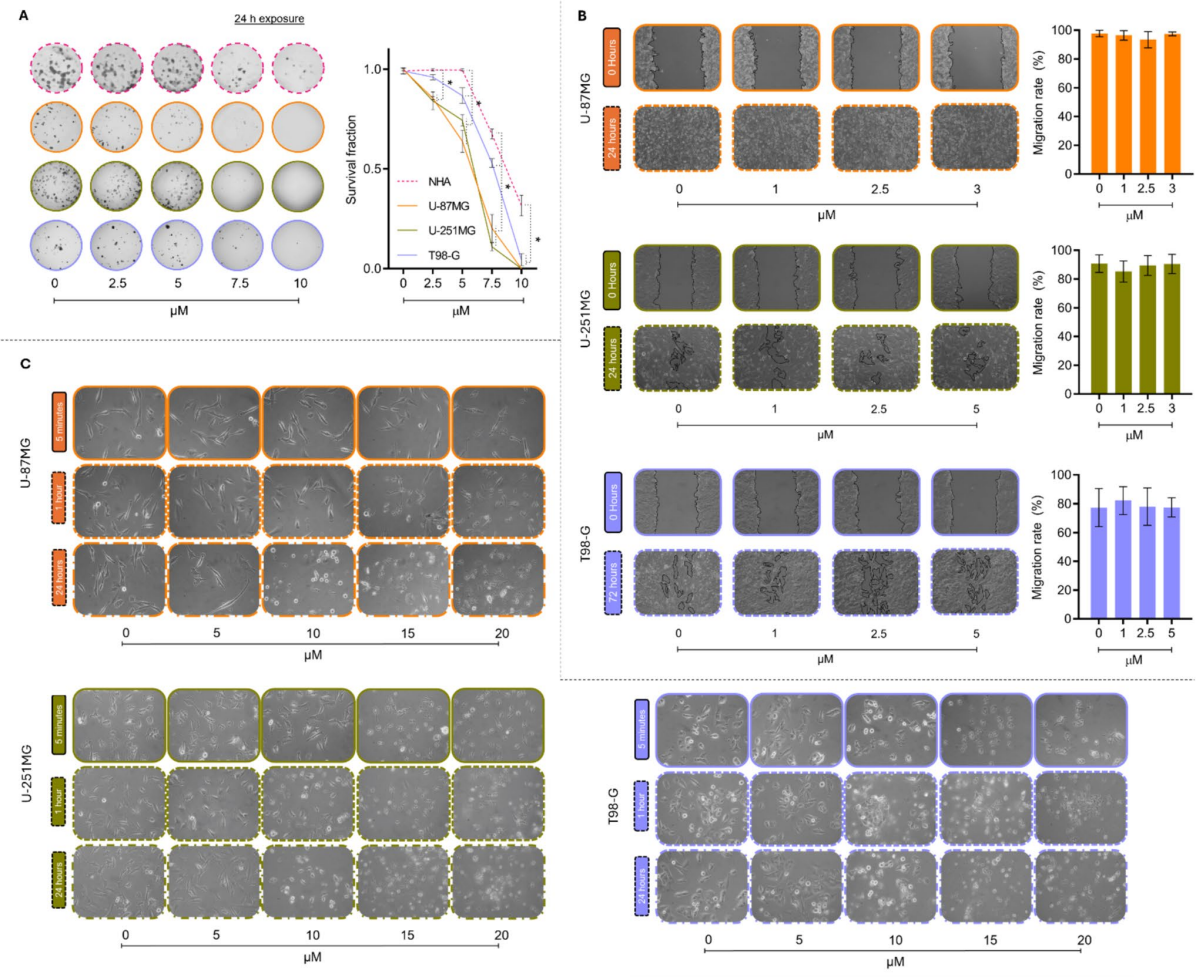
Clonogenic survival, migration and morphological alterations induced by SM in non-tumoral human astrocytes and human IDH-wildtype GB cell lines. (**A**) Clonogenic survival fraction of NHA and U-87MG, U-251MG and T98-G cells after 24 h of exposure to increasing concentrations of SM. (**B**) Representative phase-contrast photomicrographs (100 ×) and corresponding migration rates of U-87MG, U-251MG and T98-G cells after 24 h of exposure to SM. (**C**) Representative phase-contrast photomicrographs (200 ×) showing SM-induced morphological alterations in U-87MG, U-251MG and T98-G cells after 5 min, 60 min and 24 h of exposure. *SM* – Solamargine; *NHA* – Non-tumoral human astrocytes; *U-87MG* – Human glioblastoma; *U-251MG* – Human glioblastoma; *T98-G* – Human glioblastoma. Values are mean ± standard deviation (*n* = 3). *Significantly different from NHA cell line (*p* < 0.05)

The clonogenic assay revealed a clear, concentration-dependent reduction in colony formation in all cell lines exposed to SM (Fig. 2A). In U-87MG and U-251MG, concentrations of 2.5–10 µM were sufficient to significantly decrease clonogenicity compared with NHA, indicating greater long-term sensitivity of tumor cells. T98-G showed a similar but less pronounced response, with significant reductions at concentrations ≥ 5 µM. Overall, these data indicate that SM robustly suppresses the long-term proliferative capacity of IDH-wildtype GB cell lines, with sensitivity U-87MG/U-251MG > T98-G and relative preservation of NHA.

In contrast, SM did not substantially affect motility under the conditions tested (Fig. 2B)*.* In wound-healing assays, no significant reduction in migration was observed in U-87MG, U-251MG or T98-G following SM treatment, suggesting that the antiproliferative effects of SM are not accompanied by major impairment of collective cell migration in this assay.

Phase-contrast microscopy corroborated the cytotoxic and antiproliferative findings, revealing pronounced, time- and concnetration-dependent cytomorphological alterations (Fig. 2C)*.* Control cultures maintained the expected adherent, elongated phenotype with preserved cytoplasmic extensions. At 15–20 µM, early signs of injury were detectable as soon as 5 min after exposure, including edge retraction, partial loss of extensions and increased phase brightness, becoming more evident by 1 h as widespread retraction, cell rounding and an increased proportion of suspended cells. After 24 h, 5 µM SM caused only a slight reduction in confluence, whereas 10 µM led to reduced adhesion and a more contracted morphology. At concentrations ≥ 15 µM, cultures were predominantly non-adherent, with small aggregates and cellular debris, consistent with near-complete loss of viability and in line with the strong suppression of clonogenic growth.

### SM-induced oxidative stress, DNA damage, and noncanonical cell death in U-87MG cells

**Fig. 3:**
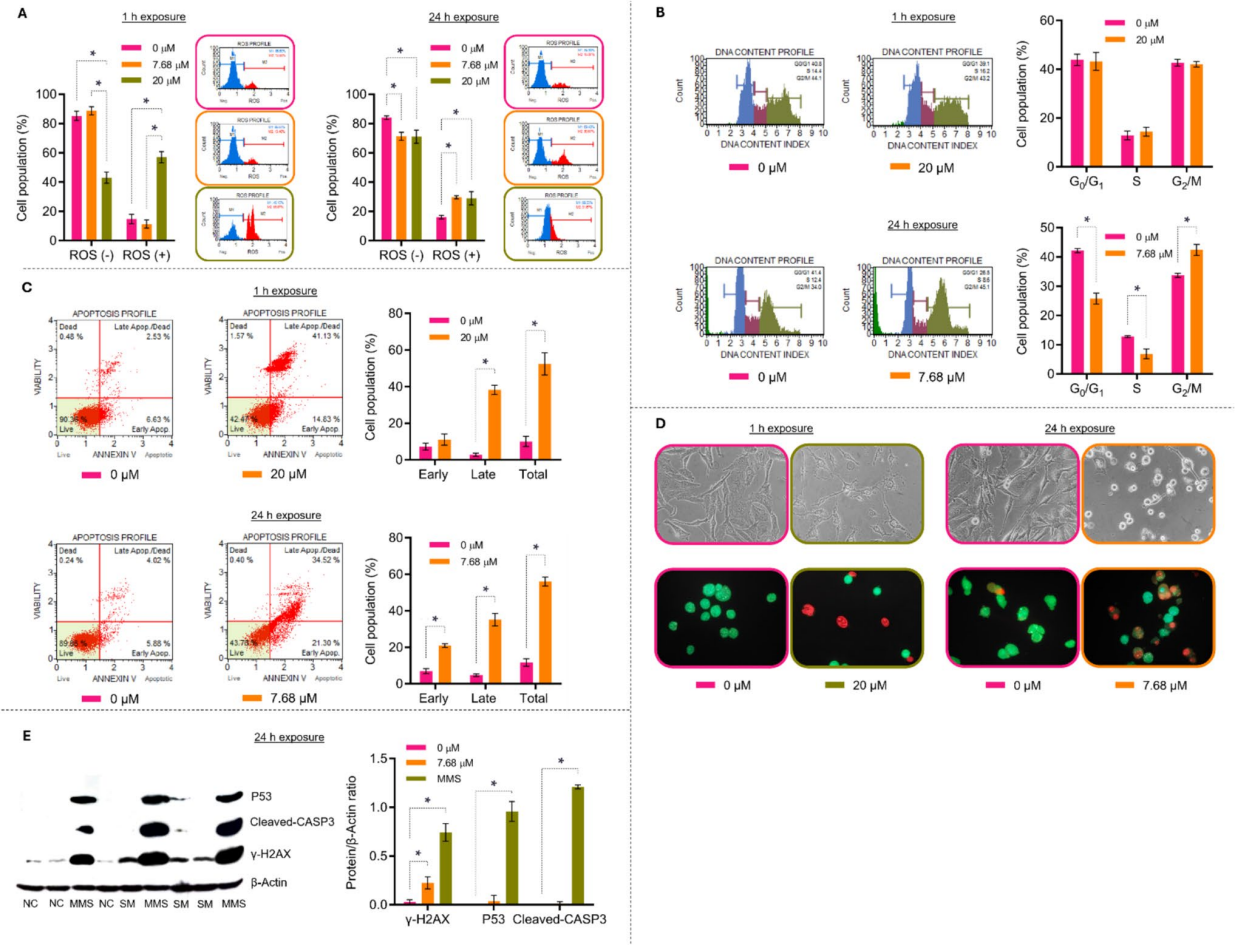
Reactive oxygen species generation, cell-cycle alterations, cell death responses, morphological changes and protein modulation induced by SM in U-87MG cells and their respective untreated controls (0 μM). (**A**) Intracellular reactive oxygen species (ROS) profile and population distribution (%) in cells exposed to 7.68 μM and 20 μM SM for 1 h and 24 h. (**B**) DNA content profile and cell-cycle phase distribution (%) of cells exposed to 20 μM SM for 1 h or 7.68 μM SM for 24 h. (**C**) Annexin V/propidium iodide (PI) staining profile and population distribution (%) of viable (Annexin V⁻/PI⁻) and Annexin V- and/or PI-positive cell subpopulations after exposure to 20 μM SM for 1 h or 7.68 μM SM for 24 h. (**D**) Representative phase-contrast and fluorescence micrographs of U-87MG cells exposed to 20 μM SM for 1 h or 7.68 μM SM for 24 h and their untreated controls. Cells were stained with acridine orange (green) to label the cytoplasm and ethidium bromide (red) to label nuclei; images were acquired at 400 × magnification. (**E**) Western blot analysis of cleaved caspase-3, p53 and γ-H2AX protein levels in cells after 24 h of treatment with 7.68 μM SM. *SM* – Solamargine; *U-87MG* – Human glioblastoma; *ROS*** –** Reactive oxygen species; *NC* – Negative control; *MMS* – Methyl methanesulfonate (1000 µM). Values are mean ± standard deviation (*n* = 3). *Significantly different from untreated controls (*p* < 0.05)

In U-87MG cells, SM triggered a concentration—and time-dependent increase in oxidative stress, cell-cycle perturbation, membrane destabilization, and DNA damage. A 1-h exposure to 20 µM SM increased ROS levels by 287% relative to control, whereas 7.68 µM for 1 h had no significant effect. After 24 h, both 7.68 µM and 20 µM significantly elevated ROS by 85% and 80%, respectively, indicating that an intense early ROS burst at high concentration is followed by a more moderate yet sustained accumulation at later time points (Fig. 3A). Consistent with an exposure-dependent effect on cell-cycle dynamics, 20 µM SM for 1 h did not significantly alter the distribution of cells in G0/G1, S or G2/M phases, whereas 7.68 µM for 24 h led to a redistribution characterized by decreased G0/G1 and S populations and accumulation in G2/M, compatible with a delay at the G2/M transition (Fig. 3B).

Annexin V/7-AAD profiling and morphology-based readouts further supported progressive membrane injury under these conditions. After 1 h at 20 µM, the Annexin V⁺/7-AAD⁻ fraction did not differ from control, but there was an increase in the Annexin V⁺/7-AAD⁺ population accompanied by a reduction in viable Annexin V⁻/7-AAD⁻ cells, suggesting early loss of membrane integrity without a clear early-apoptotic phase. At 7.68 µM for 24 h, both Annexin V⁺/7-AAD⁻ and Annexin V⁺/7-AAD⁺ fractions increased with further depletion of viable cells, indicating temporal progression of membrane destabilization (Fig. 3C). Phase-contrast and AO/EB fluorescence microscopy corroborated these findings and revealed two distinct morphological patterns: at 20 µM/1 h, cells showed irregular contours, cytoplasmic retraction and EB-positive nuclei in a subset, consistent with acute membrane damage compatible with necrotic/lytic rupture; at 7.68 µM/24 h, cells exhibited rounding, shortened processes, partial detachment, multinucleation and dual AO/EB positivity in a subpopulation, indicating gradual membrane compromise associated with cell-cycle disturbance and G2/M delay (Fig. 3D)*.*

At the molecular level, immunoblotting of U-87MG cells treated with 7.68 µM SM for 24 h showed a significant increase in γH2AX, whereas cleaved caspase-3 and P53 remained comparable to control. In contrast, the positive control MMS markedly increased cleaved caspase-3, P53 and γH2AX, confirming the sensitivity and dynamic range of the assay (Fig. 3E). Together, these data indicate that, under the tested conditions, SM induces oxidative stress and robust DNA damage signaling in U-87MG cells, with G2/M accumulation and progressive membrane injury, but without detectable engagement of caspase-dependent, P53-driven apoptosis.

## Discussion

SM demonstrated consistent antineoplastic activity in IDH-wildtype GB cell lines, reducing short-term viability and clonogenicity with selectivity over NHA and greater potency under hypoxia, whereas TMZ showed limited cytotoxicity in tumor lines and greater sensitivity in NHA.

A unifying explanation for the selectivity and increased potency under hypoxia is lectin-mediated uptake [17–21]. IDH-wildtype GB frequently displays aberrant glycosylation and increased exposure of surface glycoconjugates recognized by endocytic lectins, potentially amplified under hypoxia, which would favor carbohydrate-dependent internalization of SM [22–27]. The chacotriose group (rhamnose–rhamnose–glucose) is compatible with rhamnose-binding lectins, allowing preferential endocytosis in tumor cells, whereas non-tumor astrocytes exhibit fewer targets and more stable glycosylation, contributing to the observed therapeutic index [28, 29].

The data point to two distinct injury regimes in U-87MG. At high concentrations and short exposure (20 µM/1 h), SM triggers rapid collapse of membrane integrity, acute ROS elevation, increased Annexin V⁺/7-AAD⁺ cells and absence of cell-cycle redistribution, a pattern consistent with a predominantly lytic mechanism and poorly aligned with immediate caspase-dependent apoptosis. This profile fits the classic model of steroidal glycoalkaloids, in which the aglycone forms complexes with cholesterol and accumulation of these complexes leads to irreversible bilayer disorganization and lysis of sterol-rich membranes [30–32].

At lower concentrations and prolonged exposure (7.68 µM/24 h), SM produces a more regulated response: gradual morphological alteration, late and moderate ROS increase, G2/M accumulation, increased γH2AX and absence of cleaved CASP3 or P53 accumulation. These findings suggest activation of DNA damage and G2/M delay in a probably caspase-independent context. In light of the lectin-uptake hypothesis, a plausible scenario involves selective internalization of SM, lysosomal routing and lysosomal membrane permeabilization, releasing cathepsins and engaging lysosome–mitochondria crosstalk, culminating in mitochondrial permeabilization, secondary ROS increase and non-canonical cell death [22, 33–36]. The possibility of parthanatos (via PARP-1/PAR/AIF) is consistent with intense DNA damage and caspase-independent death, but requires direct validation with specific readouts.

Despite SM’s ability to activate multiple death programs, its combinations with TMZ were predominantly antagonistic. In U-87MG, strong G2/M accumulation after 24 h of SM reduces the S-phase fraction precisely when TMZ depends on replication to convert O^6^-methylguanine into lethal MMR-mediated lesions [37, 38]. Thus, shifting cells into a replication-poor state restricts the temporal and cellular window for S-phase–dependent TMZ cytotoxicity. Similar findings in U-251MG and antagonism in T98-G, where constitutive MGMT expression already confers additional resistance, reinforce the interpretation that the tested schedule/concentrations sequence disfavors synergy with TMZ.

While the present findings support a concentration- and time-dependent, predominantly non-apoptotic mode of cytotoxicity, further studies will be required to directly interrogate additional non-canonical cell death pathways, including autophagy-associated processes, and to validate proposed routes such as parthanatos using targeted molecular readouts. Future investigations could also refine combination strategies in light of the S-phase dependence of agents such as TMZ and directly assess determinants of selectivity and mechanism, such as lectin-mediated uptake, sterol dependence, and caspase-independent death pathways. Additionally, in vivo studies should be conducted for a better understanding of the biological effects of SM on IDH-wildtype GB.

Taken together, the results support SM as a selective antineoplastic candidate against IDH-wildtype GB, with activity maintained (or potentially increased) in hypoxic niches and concentration/time-dependent mechanisms of action: (i) sterol-associated membrane rupture at high concentration and short exposure; and (ii) a caspase-independent trajectory at low concentration and prolonged exposure, with late ROS increase, DNA damage signaling and G2/M delay.

## Data Availability

The datasets generated during and/or analysed during the current study are available from the corresponding author on reasonable request.
